# Supplementary material for: Are carbon emissions trading and green financial instruments synergistic? -Comprehensive quantitative research based on content analysis
Source: PLoS One. 2024 Mar 7;19(3):e0298601. doi: 10.1371/journal.pone.0298601 (PMC10919601; doi:10.1371/journal.pone.0298601)
Supplement: S1 Data — (ZIP) [file pone.0298601.s001.zip › Data/地级市Z_F工作报告.zip!/╕╩╦α/╞╜┴╣╩╨/└┤╫╘╣1⁄2╓┌║┼í╛┬φ┐╦╩2╛▌═°í┐_2015.docx]

各位代表：

现在，我代表市人民政府向大会作工作报告，请予审议。并请政协委员和列席人员提出意见。

2014年工作回顾

一年来，面对复杂多变的市场环境和经济下行巨大压力，在市委的坚强领导下，我们认真贯彻党的十八大和十八届三中、四中全会精神，以扎实开展党的群众路线教育实践活动为动力，牢牢把握稳中求进工作总基调和市委“深化改革增活力，提质增效促转型，统筹兼顾惠民生，凝心聚力干到位”的总体要求，全力以赴稳增长、促改革、调结构、惠民生，全市经济运行总体平稳，结构调整步伐加快，民生水平持续提升，社会保持和谐稳定。全市地区生产总值完成350.5亿元，比上年增长8%；公共财政收入24.1亿元，同口径增长19.1%，财政支出138亿元，增长2.2%；城镇居民人均可支配收入19086.1元，农民人均纯收入5395.2元，分别增长10%和12.7%。

（一）统筹推进重点领域改革，市场主体活力不断增强。我们把深化改革作为转型发展的根本动力，认真落实省、市委各项部署，从行政审批制度改革、经济体制改革、农村综合改革等关键处发力，加快转变政府职能，有效激发了市场活力。

大力简政放权，市级取消调整和下放行政审批事项119项，减少41.3%。认真做好“接、放、管”工作，对保留事项逐一明确管理层级，减要件、减程序、减环节，积极推行集中受理、一站式服务，市级31个单位183项面向公众的非涉密事项进驻政务服务中心集中办理，审批、服务行为进一步规范。完成了食药监、工商、质监管理体制调整和市级政府新一轮机构改革，事业单位分类改革稳妥推进。

认真落实促进非公经济发展各项措施，全面推行注册资本登记制度改革，新增市场主体1.26万户，全市非公经济增加值增长20%。“营改增”试点工作进展顺利，减税让利政策全面落实，有效促进了小微企业发展和创业就业。以“金财工程”信息系统为支撑的国库集中支付和公务卡应用改革全面推开，预决算公开稳步推进，支出绩效评价范围逐步扩大，依法聚财理财水平不断提升。交通银行平凉分行开业运营，平凉农商行组建工作启动实施，市投资发展公司二期企业债券发行工作有序推进，4家投融资机构实现融资26.1亿元，涉农贷款和小微企业贷款较快增长，全市金融机构贷款余额增长24.7%，金融创新能力和支持经济发展能力不断增强。

加快农村综合改革，完成了农村集体土地确权登记颁证试点，规范流转土地11.3万亩，累计达到74.3万亩。深化集体林权制度配套改革，林权抵押贷款、林下经济发展取得新的成效。水利综合改革基本完成，水价改革全面启动。支持扩大政策性农业保险，保费总额达到5567万元，增长32.4%。

（二）突出抓建“十大工程”，固定资产投资持续较快增长。我们坚持把扩大投资作为稳增长、增后劲的重要抓手，狠抓项目谋划、争取、引进和建设，全市固定资产投资增长21.4%，达到537.6亿元，拉动生产总值增长3.1个百分点。

深入研究相关区域发展规划，紧盯国家微刺激政策导向，多层次谋划对接，论证充实“3341”项目库，储备各类项目2286项，概算总投资5108亿元，其中工业项目投资占到36.8%。

围绕落实“3341”项目工程，对事关全市发展的重大项目整合打包，集中实施了能源开发等“十大工程”，以此为引领，全市实施500万元以上项目1079项，比上年增加73项，完成投资499.2亿元，增长23.9%。积极争取8个项目列入全省首批基础设施领域鼓励社会资本投资示范项目，并在全市推出66个鼓励社会资本参与建设运营的项目，吸引社会投资103亿元。

持续扩大招商引资，积极承接产业转移，先后与上海亿丰、西蒙集团、苏宁云商等大企业签订了一批合作项目，依托深圳文博会、兰洽会等平台签约项目112个，签约总额524.8亿元。强化跟踪服务，促进项目落地，实施招商引资项目391项，落实到位资金424.5亿元，增长34.2%。

（三）大力促进结构调整，产业转型升级步伐加快。我们着眼促进提质增效升级，注重发挥政策引导和市场机制作用，因势利导推进产业结构调整，加快培育现代产业体系，全市第三产业增加值增长9.1%，三次产业结构调整到24.3：38.3：37.4。

深入实施现代农业“五个百万”增收工程，创新经营模式，强化科技支撑，特色产业区域化布局、规模化发展、产业化经营势头强劲。新建改扩建龙头企业32户，新增农业专业合作社411个，新组建家庭农场61个，农业生产组织化程度不断提高。扩大肉牛冻配改良、苹果矮化密植、设施种植等先进种养技术和高效生产模式推广应用，农业科技贡献率提高到52%。完成旱作农业技术推广208万亩，粮食总产达到114.5万吨，增长3.1%；新建标准化养殖小区66个，全市肉牛饲养量、出栏量分别达到113.5万头和41.5万头；新植果园33万亩，累计达到260万亩，认证各类基地34.2万亩，果品总产达到132万吨，优果率提高到68%，“平凉金果”荣获全国驰名商标；建成蔬菜集中区15个，新增设施蔬菜1.6万亩，蔬菜总产量达到134.2万吨。

积极应对工业经济面临的严峻形势，坚持煤炭产业优化升级与壮大非煤产业同步推进，煤电主导、多元发展的新型工业化格局正在形成。大力促进煤炭产能建设和资源就地转化，《陇东能源基地开发规划》获批，《灵台矿区总体规划》重新启动申报程序，实施重大能源开发项目13个，完成投资33亿元，邵寨、赤城、五举煤矿、红河油田、20万吨聚丙烯等项目加快建设，一批矿井、火电、煤化工项目前期工作积极推进。加快新能源、新材料、装备制造和新型建材业规模扩张，光伏、碳纤维产业园一期建成投产，天纤棉业、庆华陶瓷、华煤矿机、皓天药业等项目进展良好，非煤工业占工业增加值的比重提高6.9个百分点。

着眼强化旅游商贸对三产的带动作用，大力促进文化旅游养生融合发展，文化旅游产业在经济总量中的比重逐步提高。加快实施华夏文明传承创新区“163”计划和中华崆峒养生地规划，实施文化项目55个，新培育文化企业78家，文化产业增加值增长31%。全面启动平凉旅游大景区规划和崆峒山、大云寺·王母宫景区规划编制，实施重点旅游养生项目20个，创新宣传营销方式，大型自然人文纪录片《西北望崆峒》在央视播出，举办了全国健身名山登山赛、环青海湖自行车国际比赛等节会赛事，游客接待量突破千万人次，旅游综合收入增长26.2%。陇东农副产品批发交易中心、风尚吉伍商贸建材市场、工业园区综合物流园全面建成，城乡市场体系不断完善，现代服务业较快发展，城乡消费稳定增长。

（四）积极推进新型城镇化，城乡面貌显著改善。我们坚持规划先行，完成了市域城镇体系规划初步成果、新型城镇化规划（评审稿）和第四轮《平凉市城市总体规划》修编，全市编制城乡一体化发展、村庄布局等各类规划135项，静宁县和四十里铺、安口、南湖镇列入省级新型城镇化试点县镇。

加快“一中心两园区”建设，集中实施“六提升四配套”工程，开工建设重点项目64项，完成投资71.6亿元。东大门改造二期、柳湖路东拓、东湖路及解放路改造、平沿路及太统路、青年路建设，工业园区“一纵一横”道路、南北路泾河大桥，崆峒山生态文化旅游示范区“两桥一路”等道路工程全面完成，中心城区东西畅通、南北联通的环形物流通道框架初步形成；绿地公园续建、泾河南岸景观带提升、城区雨水系统改扩建、“八沟一河”综合治理、污水收集处理、垃圾转运站建设、人行道铺砖改造、绿化带补造提升等工程积极推进；热电联产集中供热面积达到800万平方米，西北地区第一家利用水泥窑协同处理生活垃圾项目建成运行。新增清洁能源环保公交车60辆。百日集中拆违行动效果明显，城区精细化管理和居民生产生活环境质量进一步提升。抢抓政策机遇，规模化棚户区改造起步良好，新民北路东片区等重点片区房屋征收在群众支持下有效推进，全市完成房屋征收2300户、60.2万平方米，开建保障性安居工程1.2万套，建成7678套。平凉工业园区完成基础设施投资3.2亿元，新入园项目11个，崆峒山生态文化旅游示范区一批旅游服务设施项目进展良好，中心城市引领带动城镇化的能力不断增强。六县县城和重点小城镇以扩容提质和功能完善为重点，实施重点建设项目237个，建成了一批各具特色的新区、小区和片区，聚合承载能力进一步增强。

持续加强基础设施建设，天平铁路路基基本建成，平凉机场、平天、静庄、彭大高速、平华一级公路等交通项目前期工作加快推进。协调开通了火车南站集装箱业务。改造县乡道路154公里，建成通村硬化路966公里，建制村通畅率达到75%。编制完成了水资源综合规划，引洮二期静宁受水区、灵台新集水库等骨干水源工程前期工作取得较大进展，治理河堤120公里，新建农村饮水安全工程34处，解决了13.4万人的饮水安全问题。2个110千伏送变电工程进展顺利，330千伏尚家塬变电站获省发改委核准。抓建市列城乡一体化试点乡镇7个、新农村试点村20个、“三清五改”示范村200个、省列“美丽乡村”示范村14个，改造农村危房2.01万户。

以国家级生态市建设为总揽，加强生态环境保护，推进重点流域综合治理，完成造林23.6万亩，新修梯田24.4万亩，治理水土流失350平方公里。严格耕地保护，实施土地整理21万亩，代表全省顺利完成了国家土地督察西安局例行督察任务。加强节能减排、污染防治和农村环境综合整治，主要污染物排放控制在省政府下达目标值之内，区域环境质量持续好转。

（五）着力保障和改善民生，社会大局和谐稳定。我们坚持把更多的公共资源用于民生保障薄弱环节，不断完善基本公共服务制度，统筹推动社会事业改革发展，承诺措办的12件20项惠民实事完成19项，市级应急避难场所建设因规划变更计划2015年实施。全市财政用于民生的支出占到总支出的75.9%。

全面落实就业创业扶持政策，新增城镇就业3.6万人，城镇登记失业率控制在4%以内。安置应届高校毕业生8324名，就业率达到89%。发放小额担保贷款3.2亿元，带动就业1.4万人。大力开发公益性岗位，安置就业困难人员5423人。加强农村劳动力就业服务和技能培训，输转劳务49万人，创劳务收入73亿元。

着力健全社会保障体系，新农合、城镇居民医疗保险政府补助标准人均提高到320元，企业退休人员基本养老金月平均增加183.5元，失业保险金月平均提高80元，城乡低保、农村五保供养标准分别提高15%和17.7%。落实财政补助资金6039万元，启动解决崆峒区被征地农民养老保险历史遗留问题，六县被征地农民养老保险工作全面推开。实施了老年福利服务中心、社区日间照料中心等一批养老服务项目，崆峒区殡仪服务中心建成主体。

扎实推进“1236”扶贫攻坚行动，完成了贫困村、贫困人口识别和建档立卡，实施整村推进项目69个，易地搬迁群众1328户、6492人。坚持领导带头，积极内引外联扩大双联覆盖面，引进125家省内外企业联系78个贫困乡村，各帮联单位投入资金1.2亿元，实施公共性、民生性帮扶项目2120个，帮办实事1.5万件，双联行动呈现出深化拓展、重点突破、常态长效的工作格局。全市完成减贫12万人，有11个乡镇、137个村实现基本脱贫。

协调发展各项社会事业，投资7.7亿元，改扩建校舍46万平方米，新建改建幼儿园29所，华亭、崇信县义务教育均衡发展通过国家评估认定，中心城市教育布局调整步伐加快，平凉职业技术学院开工建设，平凉医专升格甘肃医学院通过全国高校专家评委会票决。推动科技创新，172项科研成果获市科技进步奖，授权专利123件。在全省率先实施了新农合分级诊疗和支付方式改革试点，启动了4个县公立医院改革试点，建成市级全科医生临床培养基地和3个县级妇幼保健机构、200个标准化村卫生室，市级儿童专科病区建设进展良好。启动实施“单独两孩”政策，为8.6万名农村育龄妇女提供了免费蓝氧治疗服务。实施了广电网络覆盖暨城域网双向升级改造，建成了642个乡村舞台和一批乡村、社区体育健身设施。启动实施了乡镇干部基本工作生活条件三年改善工程。

加强和创新社会治理，深化“六五”普法教育，大力开展平安建设“十大活动”和社会稳定风险评估，强化矛盾纠纷排查调处和信访积案化解，严厉打击各类违法犯罪活动，严密网络管理，有力维护了社会稳定。深入开展重点行业隐患排查和专项整治，安全生产形势持续稳定。强化市场监管，推进质量兴市，开展了首届政府质量奖评选表彰。健全完善食药安全监管长效机制，集中整治食品药品违法生产经营行为，确保了群众饮食用药安全。加大民族乡村扶持力度，依法管理宗教事务，“两个共同”示范区建设深入推进。统计调查、物价、档案、外事、侨务、地震、气象、人防、红十字、残疾人事业等取得新的成效。全市军政军民团结、民族宗教和顺、社会和谐文明的局面进一步巩固提升。

（六）深入开展教育实践活动，政府自身建设得到加强。我们围绕政府转职能、干部转作风，认真落实中央、省市委的总体部署和要求，聚焦“四风”突出问题，从市政府领导班子做起，扎实开展学习教育，广泛听取意见建议，带头查摆问题，抓好整改落实和建章立制，从严从实推进教育实践活动各环节工作，政府系统作风建设全面加强。市政府领导班子整改方案中确定的29个方面的重点整改任务，已整改落实24个方面，其余正在积极整改。围绕健全决策机制、密切联系群众、推动工作落实、落实党风廉政建设主体责任等方面，制定、修订各类制度37项。严格落实中央八项规定和省委双十条、市委十二条规定，大力精简会议文件，改进督查调研方式，认真开展办公用房、公务用车清理整顿，市政府会议、文件分别下降23%和16.3%，全市“三公”经费压减19.4%。扎实开展专项整治，清理取消收费项目195项，规范、清退不符合条件城乡低保对象13486人，查处各类侵害群众利益案件297起，推行农民工工资保证金制度，责令支付农民工工资1348万元，群众关注的一些热点难点问题得到有效解决。

全面推进依法行政，组建了市政府法律专家咨询委员会，健全重大行政决策公众参与、专家论证、风险评估、合法性审查和集体讨论决定制度，重视和指导各级各部门加强发展规划的制定实施，强化了对全市经济活动的引导和规范。自觉接受人大、政协监督和社会舆论监督，坚持市政府常务会议议定事项向人大报告、政协通报制度，积极配合人大、政协视察、调研活动，并认真研究和落实提出的意见建议，人大代表建议、政协提案办复率达到100%。严格落实行政执法责任制，开展了行政执法主体、行政执法人员资格清理确认，审查细化了行政处罚自由裁量权标准。扩大政府信息公开，77个市直一级部门单位全部公开了“三公”经费预决算。公共资源交易运行机制进一步完善，综合便民服务热线平台运行良好。

严格落实党风廉政建设主体责任，健全完善用制度管权、按制度办事、靠制度管人的长效机制，加强对公务人员特别是领导干部的经常性教育和监督管理，广泛开展民主评议机关作风和政风行风活动，加大纠风治乱力度，强化审计监督和行政监察，严肃查处违纪案件，政府系统廉政建设不断加强。

各位代表，一年来，全市经济社会发展取得的成绩来之不易，这是省委、省政府和市委坚强领导的结果，是市人大、市政协监督支持的结果，是全市干部群众共同努力的结果。在此，我代表市人民政府，向辛勤工作在各条战线的广大干部群众，向中央、省驻平单位和驻平部队、武警官兵，向各民主党派、工商联、无党派人士，向离退休老同志，向关心、支持平凉发展的各界人士，致以崇高的敬意和衷心的感谢!

同时，我们也清醒地看到，全市经济社会发展和政府工作中还存在不少困难与问题，主要是：宏观经济放缓及煤炭市场持续低迷、火电机组轮停调峰等因素，对我市煤电为主的工业经济造成很大冲击，加之一些重大项目推进不快，新的经济增长点培育发展较慢，致使地区生产总值、规模以上工业增加值等指标没有达到预期；城乡居民持续增收的难度加大，部分低收入群众生活还比较困难，就业和社会保障任务艰巨，教育、医疗、养老、住房、食品药品安全、社会治安等方面群众不满意的问题还比较多；政府职能转变还不到位，基层基础工作比较薄弱，服务群众“最后一公里”的问题还没有得到彻底解决，一些部门单位创新意识不强、服务效率不高、工作推行力度不大，少数干部还存在懒政怠政不作为的现象，等等。对此，我们将认真分析研究，采取切实可行的措施加以解决。

2015年工作任务

2015年是全面深化改革的关键之年，是全面推进依法治国的开局之年，也是全面完成“十二五”规划的收官之年。虽然我们仍然面临着宏观经济复苏疲软、煤电市场低迷、消费拉动增长乏力、资源环境约束趋紧等严峻挑战，但中央继续实施积极的财政政策和稳健的货币政策，进一步加大对农村和中西部地区重大基础设施建设投入，加快“丝绸之路经济带”建设，省委、省政府也在实施创新驱动战略、培育新的经济增长点、精准扶贫等方面出台了一系列政策措施，这些都为我们稳增长、调结构、惠民生提供了政策支持和有力保障。只要我们抢抓新机遇、适应新常态，坚定信心、迎难而上，就一定能推动全市经济社会迈出转型升级、科学发展的新步伐。

政府工作的总体要求是：深入贯彻党的十八大和十八届三中、四中全会精神，以邓小平理论、“三个代表”重要思想、科学发展观为指导，认真学习习近平总书记系列重要讲话，全面落实市委三届九次全委扩大会暨全市经济工作会议精神，坚持以提高经济发展质量和效益为中心，主动适应经济发展新常态，牢牢把握稳中求进工作总基调，把调结构转方式放在更加重要的位置，坚持扶贫攻坚和小康建设齐抓、城镇建设与农村发展统筹，以改善民生为根本，以提质增效为关键，以项目建设为重点，以改革创新为动力，全面加快小康和谐文明生态平凉建设进程。

经济社会发展的主要预期目标是：地区生产总值增长8%，规模以上工业增加值增长6.5%，固定资产投资增长20%，社会消费品零售总额增长12.5%，进出口总额增长10%，公共财政收入同口径增长8%，城镇居民人均可支配收入增长10%，农民人均纯收入增长12%，城镇登记失业率控制在4%以内，居民消费价格指数控制在103%以内，人口自然增长率控制在7‰以内，单位生产总值能耗和主要污染物排放完成省上控制指标。

重点抓好十个方面工作：

（一）坚定不移深化各项改革，增添转型发展新动力。着眼发挥市场在资源配置中的决定性作用，强化法治思维，运用法治方式，加快改革措施落地，促使体制机制更加顺畅、营商环境更加优越、创新创业活力竞相迸发。

深化行政审批制度改革。持续推进简政放权，做好省上下放行政审批事项承接，进一步清理下放市级行政审批事项，全面取消非行政许可审批和政府内部管理事项，加强对下放审批事项的监督管理。建立政府部门权力清单和责任清单、财政专项资金管理清单，推进政务服务网建设，推行全程代理、一站式服务和网络服务，加强公共资源交易监管，打造网上办事的并联审批平台、快捷务实的便民服务平台和信息公开的阳光政务平台。

培育壮大市场主体。全面落实支持非公经济发展政策措施，放宽准入领域，支持非公经济平等参与资源开发和国资国企改革，吸引社会资本投资文化旅游产业开发、城镇基础设施建设和公共资源配置的社会民生事业，组织实施一批社会投资示范项目。健全完善中小微企业公共服务体系，开展扶助小微专项行动，建成市中小企业服务平台和平凉工业园区小微企业创业园，启动建设市青年创业示范基地。非公经济增加值增长13%。

深化财税金融改革。推进财政预算管理体制改革，实行全口径财政预算，全面推行预决算公开，加强绩效预算管理改革。探索建立事权和支出责任相适应的财政体制，整合存量资金和部门管理专项资金，规范国库集中支付改革，严控一般性支出，集中财力保障民生支出、扶持重点产业发展。清理规范各类财税优惠政策，全面完成“营改增”任务。扩大政府购买公共服务，初步形成统一有效的购买服务平台和机制。建立政府债务管理及风险预警机制，将政府性债务全部纳入预算管理。坚持普惠金融方向，健全金融服务体系，加快平凉农商行组建进程，大力发展村镇银行。积极构建区域性资本市场，加强后备上市企业培育，设立产业引导基金，鼓励重点企业通过发行企业债、中小企业私募债等多种方式融资。加强小贷公司、担保公司、典当行和投资公司监管，打击和防范非法集资，创设良好的金融生态环境。

深化农村各项改革。建立健全土地承包经营权登记制度，加快推进土地承包经营权确权登记颁证工作，鼓励承包农户依法采取转包、出租、互换、转让及入股等方式流转承包地，探索开展农村土地承包经营权抵押贷款试点，规范土地流转行为，赋予农民更多权利和利益。加快集体林权、水资源利用、农业经营方式、社会化服务等重点领域改革，深化供销社改革，健全完善农村市场运行和农民权益保障机制。

加快国资国企改革。坚持国资改革带动国企改革，健全产权结构、法人治理结构和激励约束机制，完善以管资本为主的国资监管体系，推动国有资产监管全覆盖。建立国有资本收益收缴管理办法，规范国有资产转让行为。以规范经营决策、资产保值增值、公平参与竞争为重点，有序推进市直部门管理企业的改制脱钩，加快市属企业之间以及企业内部资源重组整合，深化交通运输、道路养护体制改革。积极发展混合所有制经济。妥善解决国有企业改革历史遗留问题。

创新园区建设管理机制。推进工业园区（集中区）及文化产业园区封闭运行、一站式服务，探索建立园区投融资公司和企业担保资金联保机制，逐步引进金融、法律、评估等中介机构，提升园区综合服务能力。在平凉工业园区、崆峒山生态文化旅游示范区积极发展“飞地”经济。启动华亭、静宁工业园区体制改革。

（二）不遗余力大抓项目建设，打造提速发展新引擎。大力推进项目带动行动，深入实施“3341”项目工程，加大招商引资力度，扩大总量，优化结构，加快培育新的经济增长点，支撑推动转型升级、科学发展。

把握政策导向谋划项目。围绕“丝绸之路经济带”、生态安全屏障综合试验区建设和陇东能源基地开发，抢抓国家实施西部地区新的差异性政策机遇，深度谋划一批支撑发展的重大基础设施项目和产业项目。加大前期投入，主动汇报对接，力促宝中铁路二线、平定兰铁路、平凉机场、引洮二期静宁受水区工程前期工作取得实质性进展，加快灵北、泾北、南川河煤矿、海洋浮力补偿系统材料等项目前期工作，争取更多项目进入国省规划盘子。

围绕转型升级引进项目。突出引“高”、引“新”、引“强”，以工业园区、产业园区为主阵地，实行网络招商、以商招商、小分队招商，推进区域性定向招商、产业性专业招商，着力引进一批战略性新兴产业和循环经济补链项目，促进产业链延伸和集群式发展。严格项目准入，严把产业政策关、投资强度关、环境保护关，推动招商引资向招商选资转变。创优投资环境，全方位搞好跟踪服务，加快项目落地建设，引进到位资金增长22%以上。

突出基础设施推进项目。建成天平铁路，开工建设平天、彭大高速、平华一级公路、平凉客运枢纽站、快速客运站，建成市道路运输应急救援指挥中心、战备桥匝道工程，争取完成静庄高速和国道312线平凉段改造前期并开工建设。加快城市配电网建设，开建750千伏六盘山至平凉输变电工程、330千伏尚家塬和110千伏灵台、泾川送变电工程，启动330千伏静宁输变电工程前期，争取国家规划建设特高压输变电工程，打通电力外送通道。实施各类水利项目60项，启动抗旱水库工程建设，加快重要河流治理和山洪灾害治理工程。全面落实“宽带中国”战略，推进“智慧平凉”建设，加快光网城市改造，实现所有乡镇通光纤，建制村无线通信和有线广播电视网络全覆盖。

强化协调服务保障项目。推行项目审批一站式服务，开辟审批服务“绿色通道”。提高重大项目决策科学化水平，加强风险评估，统筹解决项目选址、征地补偿、用水用电、企地关系协调等问题。坚持以项目定土地，强化对投入强度、产出水平等指标评估，促进未开发土地及已批未建项目用地的高效利用，积极开展城乡建设用地增减挂钩试点，保障重大项目用地需求。创新项目财政资金支持方式，引导社会资本发起设立产业投资基金和股权投资基金，支持重点项目扩大股权和债券融资，开展基础设施和公用事业特许经营。

（三）持之以恒发展现代农业，拓宽农民增收新途径。突出特色优势产业规模化、标准化、品牌化、市场化，加快推进农业现代化，促进农业增效、农民增收。

着力提升优势产业发展水平。稳定粮食生产，持续实施现代农业“五个百万”增收工程，加快标准化基地建设，健全特色优势农产品标准体系，创建粮食万亩高产示范片29个，新建标准化养牛小区20个，落实标准化果园管理措施135万亩，新增设施蔬菜1.6万亩，建成种薯扩繁基地13万亩。在突出抓好牛果菜主导产业基础上，加大种养业结构调整力度，大力发展富民多元产业、区域性特色产业，多渠道增加农民收入。培育发展家庭农场，鼓励发展多种形式的农民合作组织，推进示范社创建活动，扶持建办龙头企业，大力发展农产品加工、储藏、保鲜等业态，不断壮大新型农业经营主体。推行龙头企业加基地带农户组织模式，建立健全利益联结机制，提高农户产业收益。加大农业科技创新和新型技术集成推广力度，提高农业生产机械化作业水平。扩大农业政策性保险险种和覆盖范围，加强农业公益性服务体系建设和经营性服务组织培育，帮助农民发展生产、开拓市场。

着力加快扶贫开发进程。深入实施“1236”扶贫攻坚行动，突出政府主导，整合社会力量，推进精准扶贫和定点扶贫，实现扶贫对象精准化识别、针对性扶持和动态化管理。创新扶贫资金运作模式，积极发展扶贫资金互助社，扶持贫困户发展富民增收产业，强化新型职业农民培养，增强自我发展能力和“造血”功能。实施好65个整村推进项目，推进基础设施建设和公共服务配套，夯实贫困乡村群众稳定脱贫基础。深入开展双联行动，完善机制，整合资源，深化部门包村和村企共建活动，全面落实“六项任务”，实现干部帮扶到村到户常态化。全年完成减贫8万人，扶贫对象人均纯收入增长14%以上。

着力改善农业农村基础条件。落实最严格的耕地保护、水资源管理制度，抓好基本口粮田建设和农业综合开发。突出路、水、电、气、房，加大农村道路建设和危旧房改造力度，推进“五小水利”工程建设，加快农村电网升级改造，抓建省列“美丽乡村”示范村15个、“万村整洁村”200个，建成市列新农村试点村20个。

（四）全面推进能源基地建设，构建工业发展新格局。坚持传统产业改造升级和战略性新兴产业培育双轮驱动，着力壮大产业集群，优化工业结构，打造工业经济升级版。

加快打造千亿级煤电化冶产业集群。坚持绿色开发、深度转化、循环利用，着力提升煤化工、煤电热、煤电冶、煤电材产业聚集度。围绕煤炭、石油产业扩能增效，争取《灵台矿区规划》尽快批复，加快邵寨、赤城、五举煤矿建设，开工建设唐家河、安家庄煤矿，推进地方煤矿兼并重组，推动红河油田百万吨产能项目达产达标。加快煤电冶一体化发展，积极推动有条件企业与电厂开展直购电交易，争取实施华亭电厂二期、平凉电厂三期等火电项目，开建180万吨甲醇和70万吨烯烃、660万吨煤基新燃料项目，抓好20万吨聚丙烯项目建设。发展现代高载能产业，力争开工建设镁合金深加工循环经济一期。推进西气东输二线支线管道建设，启动中石化泾川40亿立方米煤制天然气前期工作。

促进传统产业集群式发展。实施创新驱动战略，强化企业创新主体地位，支持现有企业加快以产品结构调整为主的技术改造，培育壮大实体经济，支持康博丝特、静宁恒达等企业创建省级技术中心，大力发展高加工度、高技术含量、高附加值产品，积极培育名牌产品和名优商标，促进传统产业优化升级。启动红峰、虹光出城入园，开建平煤机高端装备制造一期、博深矿用安全设备制造项目，建成新能源汽车改装、太阳能高频无极灯项目，提升装备制造业发展水平。加快打造区域性新型建材基地，力促庆华陶瓷二期、兴旺管材生产线建成投产，积极发展节能环保石灰、脱硫石膏综合开发利用。加快实施天纤棉业二期、静宁地毯集中加工、食品速冻果蔬加工等项目，提升农副产品加工业层次。

发展壮大战略性新兴产业。合理利用和配置优势资源，完善市场化运作机制，扶持以新能源、新材料、生物产业、信息技术、节能环保、现代服务业等为重点的新兴产业快速发展。培植新兴产业骨干企业，积极推进太阳能、风能资源开发利用，力争庄浪、泾川一期50兆瓦光伏发电项目开工，促进皇甫谧制药、皓天药业、生物胶原蛋白肽生产线达产达标。抓好甲醇汽车试点推广工作。

（五）融合发展文化旅游养生，培育第三产业新亮点。以华夏文明传承创新区和陇东南国家级中医药养生保健旅游创新区为平台，以中华崆峒养生地为载体，突出商贸物流带动，加快培育新业态，推动三产发展提速、效益提升，第三产业增加值增长10%。

促进文化产业扩量提质。加强文化产业园区建设，推动文化、旅游、科技融合，打造特色文化产业集群。加快“文化集市”建设，扶持发展文化演艺、节庆会展、文体培训等基础产业，培育网络服务、创意设计等新兴产业，抓建一批文化骨干企业，着力构建文化产品交易平台。加大文化产业招商力度，抓好文化项目落地实施，文化产业增加值增长30%以上。

推进平凉大景区建设。围绕打造“神奇秀美崆峒山，天下养生第一地”和“西有敦煌莫高窟、东有泾川大云寺”的目标定位，完善大景区规划，引入市场机制，加强核心景区基础设施配套，推进崆峒山旅游配套设施二期、原生态民俗文化体验区、崆峒生态旅游度假山庄、大云寺·王母宫景区建设。启动实施乡村旅游富民工程，鼓励发展生态观光、民俗体验等新业态，规范提升农家乐，建设崆峒镇等旅游专业镇，发展一批旅游专业村。组织举办系列文化旅游节庆活动，注重运用微博微信等新媒体和信息化手段宣传推介，提高平凉旅游知名度，旅游综合收入增长22%。

大力发展养生产业。加快中华崆峒养生地建设，培育养生产业市场主体，规范国医养生馆运行，发展养生膳食、中医保健、养生用品研发制造等养生产业，推进养生主题园区、养生重点项目、养生龙头企业和养生乡村建设，健全完善现代养生产业体系。加快发展养生养老服务业，鼓励社会力量兴办养生养老服务机构。

加快发展商贸物流业。推进商贸物流中心综合体建设，加快义乌商贸城、金果博览城等商贸物流设施和县乡农贸市场建设，积极培育冷链物流、仓储配送、农超对接和鲜活农产品流通体系，畅通农产品进城、工业品下乡渠道。加快建设社区便民市场、便民超市，打造15分钟便民经济圈。创建淘宝网“特色中国·平凉馆”，引进培育电商企业，开发特色商品，鼓励企业和产业协会建立网络营销体系，推进电子商务快速发展。大力发展融资租赁、家政服务、信息中介、健康养老等生产生活性服务业。

（六）协调联动推进新型城镇化，创造统筹发展新优势。围绕打造“丝绸之路经济带”黄金段重要节点城市、省域副中心城市和甘肃东部门户城市，加快构建“四位一体”的城镇骨干体系，促进城乡一体化发展。

强化规划引领。完成市域城镇体系规划和新型城镇化规划编制，开展平凉都市区战略规划编制，构建“一主三副三节点、两轴三片双通道”的市域城镇空间格局。抓好新型城镇化试点县、镇规划和静宁、华亭县“多规合一”规划编制。完善法定规划体系，加快中心城市片区控制性详规和综合交通、市政基础设施、综合防灾等专项规划修编。围绕塑造城市风貌特色，加快编制历史文化保护规划。强化规划监管，坚决整治违法建设。

推进中心城市建设提质提速。围绕“一中心两园区”总体布局，突出“六个着力、六个突破”，实施新科路等3条续建和新民北路、和阳路等10个新建项目，加快过境车辆综合服务中心等项目建设，打通“断头路”，完善交通服务设施。加快大岔河桥头等10个片区续建和新民北路西片、砖厂新区等5个新片区的棚户区改造，实施一批便民市场、背街小巷改造、公共停车场、公厕等项目，回应群众关切，改善人居环境。抓好绿地公园两侧商业综合体续建等项目，加快盘旋路、火车站等节点改造，推进虎山公园、东湖公园和崆峒大道景观带建设，持续打造“一轴两带”城市景观，彰显城市特色，提升城市品位。加快城区新水源工程勘查建设，抓好城市供水管网维修改造、雨水系统改扩建、生活污水收集处理、集中供热、弱电管网等市政项目，实施甘沟、羊渠沟南段等9处防洪工程，完善市政设施，提升承载能力。持续推进崆峒山生态文化旅游示范区生态、文化、休闲、养生设施配套，加快平凉工业园区道路、管网、物流等基础设施建设，完善服务功能，促进产业聚集。强化城市综合执法，推进精细化管理，切实解决物业管理、交通秩序、市容市貌等方面的突出问题。

统筹城乡发展。创新城乡一体化发展体制机制，加快户籍制度改革，完善相关配套政策措施，有序推进农业转移人口就地就近市民化，实现城镇基本公共服务常住人口全覆盖，全市城镇化率提高2个百分点。推动新型政区设置，争取华亭县改市、崇信县改区，优化建制镇布局。抓好“一县三镇”省列新型城镇化试点，建设一批文化旅游、商贸集散、绿色产业城镇，抓建市列城乡一体化试点乡镇7个，推进历史文化名城名镇名村和传统村落保护利用。统筹抓好县城、重点小城镇基础设施和公共服务配套，加强绿化、净化和美化。

（七）多措并举加强生态建设，拓展持续发展新空间。实施最严格的环境监管，推动最集约的资源利用，开展最积极的生态建设，让平凉的天更蓝、水更清、山更绿、空气更清新。

更加注重节能循环。深化资源性产品价格改革，抓好民用气、自来水阶梯价格改革，加强公共机构节能管理，推进重点领域节能降耗。加快淘汰落后产能，培育循环经济优势产业链。推进华亭、平凉、静宁工业园区循环化改造，实施工业节能节水、技术改造和循环经济项目20项。推广“畜－沼－果（菜）”循环农业发展模式，加强农业废弃物循环利用。

更加注重生态保护。抓好国家级生态市建设，启动国家级环保模范城市创建。加强重点流域生态修复保护和林业生态工程建设，持续开展城镇面山和道路绿化，完成造林25万亩，新修梯田24万亩，治理水土流失220平方公里。以实施陇东百万亩土地整治项目为重点，开发整理土地23万亩。加强城乡饮用水源地保护，抓好城市中水利用和地下水源井关闭，着力解决地下水超采问题。

更加注重环境治理。健全政府、企业、公众共同参与机制，加强源头治理，严格环境准入，控制和减少高能耗、重污染项目。加快工业脱硫、脱硝、除尘改造，抓好城区扬尘、汽车尾气、餐饮油烟污染治理，扩大集中供热覆盖面，淘汰燃煤小锅炉，持续改善大气环境。加强水污染防治，开建平凉、华亭工业园区工业污水集中处理工程，推进以农膜回收利用、畜禽粪便处理为重点的农村面源污染治理，强化固废危废管理，加强重点行业、重点企业环境执法监管，确保污染物达标排放。

（八）补短兜底保障改善民生，共享改革发展新成果。加大民生投入，促进财力向民生倾斜、政策向民生聚焦、服务向民生覆盖。

集中力量措办惠民实事。1.改造县乡道路100公里，建设通村硬化路1000公里。2.新建农村饮水安全工程26处，解决3.7万人的饮水安全问题。3.新建保障性安居工程9459套（户）；完成农村危旧房改造1.3万户。4.新建、改扩建校舍10万平方米，建成标准化学校300所。5.新建标准化村卫生室30个；建成10个乡镇卫生院职工周转宿舍。6.新建市博物馆；建设龙隐寺公园；建成“乡村舞台”400个。7.建成平凉体育健身广场；新建村级体育健身工程100个；面向社会开放平凉十中、医专等学校体育运动区。8.全面推开城乡居民大病医疗保险；建立临时救助制度。9.完善法律援助便民机制，扩大援助覆盖面，形成“城镇半小时”、“农村1小时”法律服务圈。10.建成市残疾人康复中心；实施“十大助残民生项目”。

积极推进创业就业。全面落实就业政策，健全完善创业就业支持和服务体系，多渠道开拓就业岗位，新增城镇就业3.3万人。把高校毕业生就业放在首位，积极引导毕业生转变就业观念，坚持市场配置与政府购买岗位安置相结合，实施公开招录聘用、民生实事就业、基层服务、“四支”招募计划，普通高校毕业生就业率保持在85%以上。加大就业援助力度，扩大小额担保贷款覆盖范围，动态消除“零就业”家庭。加强劳务培训和技能鉴定，维护农民工合法权益，鼓励引导农村劳动力就地就近转移就业。

努力提升社会保障水平。扩大社会保障覆盖范围，推进社保信息化建设，稳定提高养老、医疗、工伤、失业保险待遇水平。探索城乡居民基本医保制度整合，完善医保异地就医和费用结算管理。做好失业保险和工伤保险省级统筹工作。落实被征地农民养老保险制度。加强城乡低保动态管理，提高城乡低保、农村五保和城市“三无”老人供养补助标准，建立社会救助和重度残疾人护理补贴、贫困残疾人生活补贴制度。

协调发展社会事业。促进教育优先发展、均等发展，深化教育领域综合改革，实施专项教育保障能力建设，加快推进“全面改薄”项目，重视办好农村小规模学校。优化调整城区学校布局，新建一批标准化中小学校和幼儿园，合理配置教育资源，着力解决择校热、大班额、入园难的问题。泾川、灵台县通过义务教育均衡发展国家评估验收。加快医专升本和职业技术学院建设。突出中医特色，深化医药卫生体制改革，加强医疗服务和公共卫生服务能力建设，全面推进分级诊疗，实施重大疾病防控、乡镇卫生院附属工程等建设项目，加快市级儿童专科病区建设。持续加强人口和计生工作，稳定低生育水平，有效落实奖励优惠政策和“单独两孩”政策。加大科技经费投入，鼓励科技创新，推进产学研结合，建成市科技孵化器。分类推进文广事业单位改革和经营性文化单位改革，建成宝塔公园，组织办好市第四届运动会和全国武术比赛，积极扶持发展体育产业，鼓励支持全民健身运动。深入开展精神文明创建活动，传承弘扬中华民族优秀传统文化，实施“24字人信人守工程”，培育和践行社会主义核心价值观，提升全民道德素质和社会文明程度。

（九）加强和创新社会治理，凝聚和谐发展新合力。深入开展法治宣传教育，推进公共法律服务体系建设，增强全民法治观念。积极推动社会治理方式创新，推进平安建设，构建立体化社会治安防控体系，提高重点人群管理服务水平，强化网络监管，加大防范和打击暴力恐怖活动工作力度，依法打击违法犯罪活动，妥善处置群体性事件。健全重大事项社会稳定风险评估、化解矛盾纠纷综合调处和维稳应急处置机制，推行诉访分离、逐级走访、网上信访，建立涉法涉诉信访依法终结制度，有效预防和减少不安全不稳定因素。坚持管理重心下移，加强城乡社区建设和政法基础设施建设，努力改善基层干部基本工作生活条件。严守安全生产红线，强化打非治违和隐患排查治理，严格目标管理与责任追究，杜绝重特大生产安全事故。健全基层监管体系，加大食品药品安全日常监管和专项整治力度，保障人民群众饮食用药安全。加强政府质量工作，不断提高产品质量、工程质量、环境质量和服务质量。加强防灾减灾机制和体系建设，提高灾害防御能力。推进诚信建设制度化，提高失信成本，倒逼诚信体系建设。依法规范宗教事务，支持民族乡村加快发展。深化双拥工作，创建“全国双拥模范城”。

（十）高质量编制“十三五”规划，谋划科学发展新蓝图。着眼长远发展，顺应人民期盼，注重衔接2020年全面建成小康社会各项目标，研究编制好“十三五”规划，引领和促进全市经济更有效率、社会更加公平、发展更可持续。围绕产业转型升级、调整优化城乡结构、促进区域协调发展、加强社会建设、全面深化改革、推进依法治市等方面，做深、做细39个重大课题前期研究，尽快形成高质量的调研成果。突出战略性、纲领性、综合性，研究把握“十三五”期间宏观经济走向，确定提出全市国民经济和社会发展总体目标任务，合理制定指标体系，明确优势产业发展方向、重大建设工程和重点项目布局、保障和促进发展的主要措施，3月底前形成规划纲要框架，9月底前完成纲要草案。同时，组织编制好确定的61个专项规划。2016年2月底前，全面完成各类规划的编制、审批和发布实施。编制“十三五”规划是一项宏大的系统工程，需要各级各部门各司其职、密切配合、协同推进，更需要充分发扬民主、集思广益、问计于民，希望各位人大代表、政协委员及社会各界人士积极建言献策，使规划编制工作成为汇聚民智、凝聚共识的过程，形成群策群力谋划新蓝图、齐心协力开启新征程的强大合力。

加强政府自身建设

各位代表，全面深化改革、加快转型发展、推进依法治国对政府工作提出了新的要求。我们将认真贯彻党的十八届四中全会精神，以建设法治政府为目标，按照省、市委“工作落实年”的要求，着力提高政府执行力、公信力和依法行政能力，更好地担当起推进全市改革发展的重任。

坚持依法行政。自觉运用法治思维、法治办法，审视发展改革问题，破解发展改革难题。按照法定权限行使权力，严格执行重大行政决策法定程序，发挥法律专家咨询委员会作用，进一步提高行政决策的民主化、科学化水平。优化调整执法机构，严格实行行政执法人员持证上岗和资格管理制度，促进执法重心和执法力量向基层下移，探索有条件的领域推行跨部门综合执法。建立健全行政裁量权基准制度，完善执法程序，全面落实行政执法责任制和过错追究制，提高执法效率和规范化水平。

坚持务实勤政。巩固扩大教育实践活动成果，认真落实整改任务，深入推进专项整治，严格落实改进作风各项规定，持之以恒纠正“四风”问题，驰而不息正风肃纪。大力倡导求真务实作风，切实加强和改进调查研究，接地气、查实情、出实招，增强指导和推动工作的针对性、有效性。健全决策目标、执行责任和考核监督体系，创新督查机制，改进督查方式，强化绩效考评，以目标倒逼进度，以督查促进落实。严格执行首问负责、限时办结、公开承诺、责任追究制度，坚决纠正懒政怠政不作为的问题，敢于直面矛盾，勇于攻坚克难，尽心尽力，主动作为，激情干事，勤以治事，全面提升服务效能。

坚持廉洁从政。严格落实党风廉政建设主体责任，扎实推进惩治和预防腐败体系建设。加强对工程建设、资源开发、土地管理、公共资源交易、政府采购等重点领域的行政监察和审计监督，推进公共资金、国有资产、国有资源和领导干部履行经济责任情况审计全覆盖，严肃查处违纪违法案件。认真落实厉行节约反对浪费各项规定，大力倡导艰苦奋斗、勤俭节约的作风，切实加强公务接待、公务用车管理，严格控制“三公”经费支出。

坚持阳光施政。认真执行人大及其常委会的决议决定，主动接受人大法律监督、工作监督和政协民主监督，完善协商民主制度，积极开展协商民主试点工作，不断提高人大代表建议和政协提案办理质量。认真听取社会各界的意见建议，自觉接受社会舆论监督。全面深化政务公开，完善新闻发布制度，加强电子政务建设，提升平凉门户网站集群建设管理水平，加大与人民群众息息相关的办事程序、收费标准、补贴政策、规定依据等公开力度，推进公共资源配置、重大项目实施、社会公益事业建设等领域政府信息公开，保障人民群众知情权、参与权和监督权。

各位代表，改革激发活力，法治引领发展。站在全面深化改革、加快转型升级的新起点上，让我们在省委、省政府和市委的坚强领导下，紧紧依靠全市各族人民，以更加奋发有为的进取精神，更加勤勉扎实的工作作风，开拓创新，奋勇争先，为全面完成“十二五”规划各项目标任务、加快建设小康和谐文明生态平凉而努力奋斗！
